# The double face of mitochondrial dysfunction

**DOI:** 10.18632/aging.100923

**Published:** 2016-03-05

**Authors:** Dmitry Knorre, Anna Zyrina, Fedor Severin

**Affiliations:** Belozersky Institute of Physico-Chemical Biology, Moscow State University, Moscow, Russia

**Keywords:** mitochondria, cancer, aging, yeast, mtDNA, checkpoint

Aging is associated with increased manifestation of many diseases. In particular, the chances of cancer increase exponentially starting from middle age. At the same time, there is a convincing evidence that in the case of certain tissues/organs the increase of cancer incidence is followed by a plato or even a decrease of reported cases [1]. As a rule, the decrease starts at the age above 80 years. What could be the reason for such a decline? Possibly, only a small proportion of human population is susceptible to the certain types of cancer and most of such people die at the age of 80 or younger. If so, the age group above 80 will be depleted for people prone to the cancers. Alternatively, several biological explanations for such a decrease were suggested (see [1]): for instance, age-dependent decrease of angiogenesis can suppress tumor growth.

We propose that mitochondrial dysfunction is also responsible for the age-dependent changes of cancer incidences. First, mitochondrial oxidative capacity and ATP production decrease with age [2]. The reasons for the mitochondrial dysfunction are the mutations of mitochondrial DNA (mtDNA) and a continuous expansion of mtDNAs with large deletions. Second, in aged mammals the clonal expansion is likely to be a primary source of abnormal mtDNAs rather than de novo mutations [3]. How could mitochondria dysfunction interfere with cancer incidence and progression? On one hand, mild mitochondrial dysfunctions can be cancerogenic due to increased mitochondrial reactive oxygen species-mediated inflammation (see [4]) or due to inhibition of apoptosis [5]. On the other hand, the severe ones can inhibit the proliferation of cancerous cells. Cruz-Bermudez et al. [5] have shown that the cells lacking mtDNA have lower tumorogenic potential compared to the ones with the mild mutations or even with intact mtDNA. This is surprising, because for their energy needs cancer cells tend to rely on glycolysis instead of oxidative phosphorylation. Our study with model eukaryotic cells – baker's yeast – provides an explanation: we found that the loss of mtDNA activates a signaling cascade that tightens the S-phase arrest of the cells caused by inactivation of telomerase [6]. Importantly, this effect was not due to alterations in ATP supply. We speculate that such mitochondria-dependent signaling pathways play a significant role in the regulation of cell cycle progression in higher eukaryotes.

If so, it provides an explanation for the age-dependency of cancer incidences. During the development and aging of multicellular organisms various mutations appear in mtDNAs. Some of them have severe tumorogenic capacity and eventually lead to cancerous transformation of the host cells. We speculate that, with age, the severe mutations (i.e. common deletions of mtDNA) expand and prevail over the mild ones, strengthening the S-phase arrest and thus decreasing cancer incidences. Interestingly, this hypothesis explains why, during evolution, mtDNA of some higher eukaryotes (including humans) did not get rid of the repeat regions which are prone to recombination leading to the common deletions. In other words, the intrinsic instability of mtDNA may serve as a cancer-prevention mechanism. Apparently, among all types of cancer, the long-living (transmissive) ones are likely to be most sensitive to such instability. Indeed, it was shown that canine transmissible venereal tumor undergoes several rounds of mtDNA acquisition from the host cells [7].
